# Influence of the Initial Guess on the Estimation of Knee Ligament Parameters via Optimization Procedures

**DOI:** 10.3390/bioengineering11121183

**Published:** 2024-11-22

**Authors:** Ilias Theodorakos, Michael Skipper Andersen

**Affiliations:** Department of Materials and Production, Aalborg University, 9220 Aalborg, Denmark; msa@mp.aau.dk

**Keywords:** knee joint, laxity, optimization, ligament parameters, multibody modeling, force-dependent kinematics

## Abstract

Optimization procedures provide ligament parameters by minimizing the difference between experimental measurements and computational simulations. Literature values are used as initial guesses of ligament parameters for these optimization procedures. However, it remains unknown how these values affect the estimation of ligament parameters. This study evaluates the effects of the initial guess on estimations of ligament parameters. A synthetic data set was generated using a subject-specific knee computational model, reference ligament parameters and simulated laxity tests. Subsequently, ligament parameters were estimated using an optimization routine and four different initial guesses. The distance of these initial guesses from their true values ranged from 0 to 3.5 kN and from 0 to 3.6% for the stiffness and reference strains, respectively. The optimized ligament parameters had an average absolute mean error ranging from 0.15 (0.09) kN and 0.08 (0.04)% to 3.67 (2.46) kN and 1.25 (0.76)%, while the kinematic error remained below 1 mm and 1.2° for all conditions. Our results showed that the estimations of the ligament parameters worsened as the initial guesses moved farther away from their true values. Moreover, the optimization procedure resulted in suboptimal ligament parameters that provided similar behavior to the true laxity behavior, which is an alarming finding that should be further investigated.

## 1. Introduction

Subject-specific musculoskeletal models are commonly used to estimate joint, muscle and ligament forces, which assist in addressing orthopedic- [1,2] and injury-prevention [3,4]-related research questions. Frequently, ligamentous structures are incorporated into such models, as their function is important for the biomechanical behavior of the joints [5]. The needed material properties are adopted from generic/average literature-based values, originating from mechanical tests performed on cadaveric specimens [6,7]. However, it has been demonstrated that computational models provide closer agreement to experimental measurements when subject-specific material properties are employed [1,2]. As such values cannot be measured directly in a non-invasive manner, methodologies have been developed to provide estimations of ligament material properties in an indirect manner [1,2]. These procedures combine experimental measurements, computational simulations and optimization routines [1,2]. The ligament parameters are computed using optimization procedures that minimize the difference between computationally simulated and experimentally measured joint kinematics resulting from laxity tests. A laxity test measures the passive kinematic response of a joint under the influence of an externally applied load. Such tests are commonly used in clinical practice to determine the integrity of ligaments [8] and in biomechanical research to help in obtaining subject-specific ligament parameters [1,2]. Several studies have employed such methods [1,2,9,10,11] and they have reported different levels of agreement between experimental and simulated behaviors due to the experimental protocol, the complexity of the model and possibly the kind of the optimization procedure.

Blankevoort and Huiskes [11] presented a workflow to optimize the reference strains of the anterior and posterior cruciate ligaments (ACL, PCL) and medial and lateral collateral ligaments (MCL, LCL). Experimental laxity measurements on anterior–posterior translation and internal/external rotation for different knee flexion angles were used. The authors reported better agreement between the simulations and the experimental laxity when the optimized reference strains were used compared to the non-optimized ones. Later, Baldwin et al. [1] used internal/external and varus/valgus laxity tests to obtain subject-specific reference strains and stiffnesses for soft tissue structures crossing the knee joint via an optimization procedure. Simulations with the optimized parameters demonstrated smaller kinematic differences from the experimental values compared to using generic literature values. The model with the optimized properties resulted in a root-mean-square error (RMSE) of 2.6 (0.3)° for internal rotation and 1.4 (1.0)° for valgus, compared to 9.2 (4.8)° and 3.6 (2.7)° when using the literature generic values. Similar procedures were used by Ewing et al. [2] to estimate soft tissue properties in knees after Total Knee Arthroplasties (TKA). However, they reported worse kinematic agreement (RMSE of 4.3 (2.9)° for varus/valgus and 3.2 (2.2)° for internal/external rotation) compared to previous studies, possibly due to the fewer experimental trials employed.

These studies fitted their models to experimental data sets and computed ligament parameters via optimization procedures. The authors used, as initial guesses of the optimization process, values that were adopted from tensile tests conducted on cadaveric specimens [6,7]. Moreover, during the optimization process, the parameters were bounded within the existing experimental literature values [1,2]. However, these literature values were adopted from studies with small sample sizes. The study by Butler et al. [6] employed three specimens (two females, ages ranging from 21 to 30 years old), while Trent et al. [7] performed tensile tests on six specimens from a subset of 10 cadaveric knees with ages ranging from 29 to 55 years old. These studies most likely do not represent the existing population variability. Therefore, the employed initial guesses and the imposed bounds in the optimization processes limit the procedure from finding the true ligaments parameters for every individual. The optimization problem of estimating ligament parameters is a non-convex problem, possibly with many local minima, at least when models that include multiple ligamentous structures with nonlinear behavior are employed. Thus, it is possible that the procedure will provide a suboptimal solution, close to the provided initial guess, that results in similar biomechanical behavior to the true behavior. Models with inaccurate ligament parameters could lead to suboptimal designs or to misleading conclusions about interventions. It is, therefore, important to evaluate whether the initial guess of the optimization procedure influences the estimations of the ligament properties and, if so, how.

Recently, a case study was presented where two different sets of knee ligament parameters were obtained via optimization procedures [12]. This occurred because two different initial guesses were used: a generic set of values adopted from the literature and another with subject-specific values for ligament stiffnesses obtained from tensile tests. The results showed that the initial guess could affect the obtained ligament parameters. However, the study relied on experimental measurements susceptible to uncertainties, which influence the estimation of ligament parameters [13]. Therefore, this article, which is a revised and expanded version of a paper entitled “Influence of the Experimental Protocol and the Optimization Method on the Noninvasive Estimation of Knee Ligaments Properties”, which was presented at the International Conference on Digital Human Modeling, Antwerp, Belgium, in 2023 [14], aims to evaluate how the initial guess of an optimization routine affects ligament parameter estimation using a computationally generated synthetic data set, free of experimental inaccuracies and uncertainties. We hypothesized that the further away the initial guess is from the reference values, the worse the estimation of the ligament parameters will be.

## 2. Materials and Methods

### 2.1. Experimental Data

The experimental data were obtained from a previous study [15], but are briefly described here for completeness. A 25-year-old healthy male (height 181 cm, mass 72 kg) was subjected to magnetic resonance imaging (MRI) scans. His right lower limb was scanned from pelvis to foot using a T1-LAVA-Flex coronal plane scanning sequence with 1.6 mm slice thickness on a 1.5 T OptimaTM MR450w—70 cm scanner (General Electric Healthcare, Chicago, IL, USA). Three overlapping scans were obtained to cover the full limb, and General Electric software was used to stitch them together. The first scan started at the top of the pelvis, the second targeted the knee joint and the third ended containing the lower end of the calcaneus bone. A detailed MRI scanning sequence, following the OAI knee protocol [16], was performed on the knee joint complex to obtain the geometry of the articular cartilages. The procedures were approved by the North Denmark Region Committee on Health Research Ethics (N-20180077).

The femur, tibia and fibula bones were manually segmented using the full limb scans, while the articular cartilages were segmented from the detailed knee scans. The digitalized representations of the bones and their cartilages were exported as stereolithography (STL) surfaces. Mimics Research 19.0 (Materialise NV, Leuven Belgium) software was used for all segmentations.

The locations of the ACL, PCL, MCL and LCL insertion sites were identified on the segmented bones using the MRI scans. Moreover, the following anatomical landmarks were identified on the segmented bones: the center of the femoral head, medial and lateral femoral epicondyles, and medial and lateral malleoli. The ankle center was defined as the midpoint of the medial and lateral malleoli.

### 2.2. Computational Model

**Figure 1:**
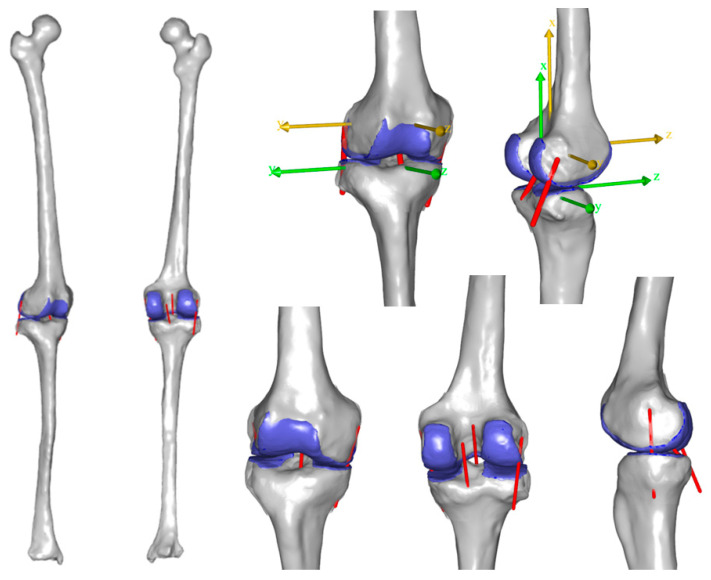
The tibiofemoral computational model. The implemented ligaments are represented by red lines. The articular cartilage is represented by blue color. The anatomical reference systems of the femur and the tibia are represented by yellow and green colors, respectively.

A computational model of the tibiofemoral joint was developed in the AnyBody Modeling System (AMS) v. 7.4 (AnyBody Technology A/S, Aalborg, Denmark). The joint included the tibial segment, the femoral segment and their articular cartilages (Figure 1). The ACL, PCL, MCL and LCL were implemented into the model as single line elements that connected their respective insertion sites. An anatomical coordinate system was defined for each segment (Figure 1) using the selected anatomical landmarks, following the work by Grood and Sunday [17].

The femoral segment was fixed in space while the tibial segment was constrained with respect to the femur at a predefined flexion angle. The remaining five degrees of freedom (DOF) (3 translations, internal/external and varus/valgus rotations) were modeled as Force-dependent Kinematics (FDK) DOF [18]. This enabled the computation of movements using the equilibrium among the contact forces generated between the articular surfaces, the forces generated by the ligaments and the externally applied loads.

Two rigid-to-rigid STL-based contact models, representing the lateral and the medial sides of the tibiofemoral joint, were defined to simulate the interaction between the articular surfaces in terms of contact forces. These contact forces were computed as the sum of the forces $F_{i}$ exerted by each vertex i that was in contact with a triangle of the opponent contact STL surface. The force of each vertex in contact was computed using the following relationship:

(1) $$F_{i}=P\;V_{i}$$

where *P* is a pressure modulus equal to 10 GN/m^3^. This nonphysical quantity has been used before for similar models [13,19] and it ensures that the penetration into the contact surfaces remains in the same order of magnitude as the kinematic tolerance. $V_{i}$ is the penetration volume of the *i*-th vertex approximated by the equation

(2) $$V_{i}=A_{i}\;d_{i}$$

where $d_{i}$ is the penetration depth of a the *i*-th vertex into the closest triangle of the opponent STL surface, and $A_{i}$ is the area of the opponent triangle.

A nonlinear force–strain relationship was used to describe the force (*f*) exerted by each ligament [11]:

(3) $$f\left(\varepsilon \right)=\left\{\begin{array}{c} k\left(\varepsilon -\varepsilon_{1}\right),\;\;\;\;\;\;\;\;\;\;\;\;\;\;\;\;\;\;\;\;\;\varepsilon >{2\varepsilon }_{1}\\ \frac{k\varepsilon^{2}}{4\varepsilon_{1}},\;\;\;\;\;\;\;\;\;\;\;\;\;\;\;\;\;\;\;\;\;\;\;\;0\leq \varepsilon \leq 2\varepsilon_{1}\\ 0,\;\;\;\;\;\;\;\;\;\;\;\;\;\;\;\;\;\;\;\;\;\;\;\;\;\;\;\;\;\;\;\;\varepsilon <0 \end{array}\right.$$

where *k* is the ligament stiffness, and $\varepsilon_{1}$ (=0.03) is a constant related to the transition toward the linear region of the *f-ε* curve. Each ligament reference length $l_{r}$ was defined from its insertion and origin sites in the MRI scans. The slack length $l_{0}$ of the ligaments was computed from the equation

(4) $$l_{0}=\frac{l_{r}}{\varepsilon_{r}+1}$$

where $\varepsilon_{r}$ is the reference strain. The ligament strain *ε* was computed using the ligament length *l*:

(5) $$\varepsilon =\frac{l-l_{0}}{l_{0}}$$

### 2.3. Model Evaluation

An evaluation of the model was performed to verify that the laxity behavior of our computational model remains within the physiological range. This was achieved by simulating laxity tests with loading conditions similar to those in previously reported experimental tests [20,21,22,23,24]. The computational model with ligament parameters adopted from the literature [25] (Table 1; reference values) was placed at 0, 20, 30 and 60° knee flexion angles, and the following loads were applied on the tibial segment: (i) 134 N of anterior force, (ii) 100 N posterior force, (iii) 5 Nm of internal and external rotation moment, (iv) 10 Nm of varus moment and (v) 8 Nm of valgus moment. The kinematic response of our model to these loads was compared against the respective literature values [20,21,22,23,24].

### 2.4. Generation of Synthetic Data/Reference Simulations

Reference simulations were conducted with the computational model using the ligament parameters displayed in Table 1 (the reference values were used). An experimental protocol similar to the one employed by Baldwin et al. [1] was used. The trials of the synthetic data set consisted of varus/valgus (VV) and internal/external rotation (IE) laxity tests, simulated by applying pure moments on the tibial segment. These moments varied in magnitude from −10 Nm to 10 Nm in steps of 2 Nm. The procedure was repeated for three knee flexion angles: 0°, 30° and 60°. The resulting knee translations and rotations were computed according to Grood and Sunday [17].

The geometrical representations of the bones, their articular surfaces and ligaments combined with the loading scenarios, and the resulting knee kinematics comprised our synthetic data set.

### 2.5. Optimization of Ligament Properties

An optimization procedure enabled the computation of the ligament parameters using the reference laxity tests as the target biomechanical behavior and a different set of ligament parameters as the initial guesses each time. The initial guesses were gradually moved farther away from the reference ligament parameters used to provide the synthetic data (Table 1). These variations in the initial guesses were used to provide an evaluation of the influence of the initial guess on the estimation of the ligament parameters.

For each of the initial guesses, the optimization procedure provided a vector *d* = [$k_{i},\;\varepsilon_{ri}$] for *i* = 1, …, 4, where $k_{i},\;\varepsilon_{ri}$ represents the stiffness and reference strain of the *i*-th ligament included in the model that minimized the difference between the simulated laxity kinematics and the reference kinematics in a least-square sense:

(6) $$\underset{d}{\min\frac{1}{2}}\sum_{j=1}^{n}\sum_{q=1}^{6}{\left(Q_{q}^{sim}-Q_{q}^{ref}\right)}_{j}^{2}\;subjected\;to\;\;\;\;\;\;\;\;\;\;\;\;\;\;\;\;\;\;\;\;\;\;\;\;\;\;\;\;\;\;\;\;\;\;\;\;\;\;\;\;\;\;\;\;\;\;\;\;max(F_{j}^{FDK})<0.001\;\;k_{i\;}\geq 0,\;i=1,\;\ldots ,\;4$$

where $Q_{q}^{sim}$ and $Q_{q}^{ref\;}$ are the simulated and the reference *q*-th generalized coordinate of the knee joint, representing the three knee joint translations in mm and the three knee joint rotations in °. The number of laxity tests n is 63. $F_{j}^{FDK}$ is the FDK residual force and moment for the *j*-th laxity trial.

The Complex optimization method [26] was selected to solve the problem described in Equation (6). The Complex optimization is a direct method and, although it is more computationally expensive compared to gradient-based methods, is efficient and convenient in optimizing problems with nonlinear objective functions. The method is based on the Simplex method [27], but it uses more points than the Simplex during the search process, which contributes towards converging to better local minima, thus avoiding premature termination. This method has been successfully used for many applications, such as calibrating macroscopic traffic flow models [28], solving the iterative closest point problem [29] and astronomical image-fitting [30]. The algorithm was implemented into the optimization process as described in [13]. For each optimization process, a population of 20 candidate solutions was randomly generated around the initial guess of the ligament parameters in intervals of ±2 kN and ±3% of the given initial guess for the stiffness and the reference strain, respectively. This procedure was selected to reduce the risk of the algorithm being trapped in nearby suboptimal local minima. A value equal to 0.9 was selected for the reflection factor, as preliminary simulations showed faster convergence compared to using values higher than 1. If the candidate solution with the worst objective value remained the same after four attempts, the candidate solution was replaced by a new random one and the algorithm was reset. Different initial guesses were adopted to simulate the real-life experimental challenge where the true ligament properties values are not known a priori. Table 1 summarizes the different initial guesses that were used for the ligament parameters. All trials were solved in AMS using the FDK solver in a single step. The AnyPyTools library [31] was used to enable parallel processing of the trials.

The differences between the optimized and the reference ligament parameters were calculated. Subsequently, the effect of the initial guess on the optimization process was quantified by evaluating the averaged stiffness differences and the averaged reference strain differences over all ligaments for each initial guess. Furthermore, the RMSE between the reference kinematics and the kinematics obtained from the optimized ligament values was calculated.

## 3. Results

**Figure 2:**
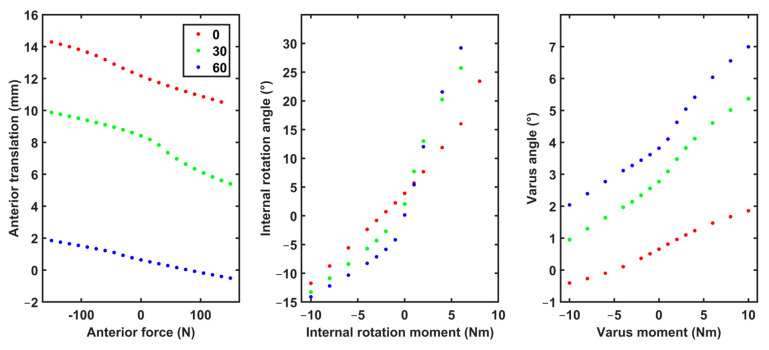
The laxity profile of the synthetic data generated using the computational model and the reference ligament parameter.

The comparison between the simulations with our model and experimental measurements from the literature [20,32] showed that the laxity profile of our model (Figure 2) was within the range of previously reported physiological values (Table 2).

**Figure 3:**
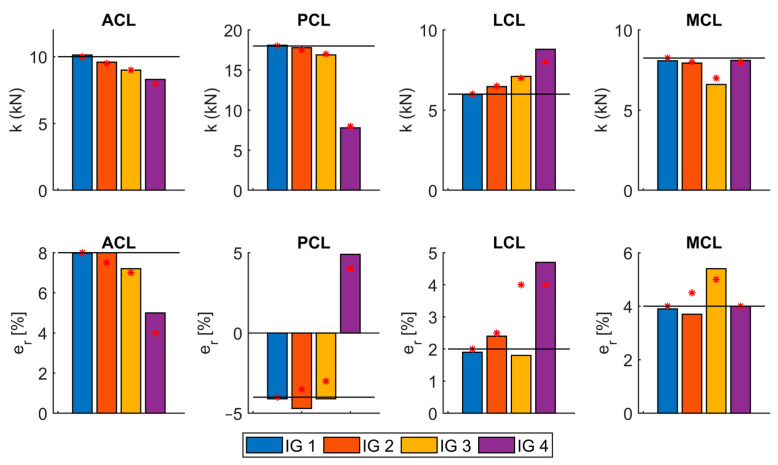
Optimized ligament parameters for different sets of initial guesses (IGs). The red asterisks show the respective initial guess values, while the black line represents the reference values.

The error of the estimated ligament parameters increased as the initial guess moved farther away from the reference values. The mean stiffness and reference strain error of the ligaments increased from 0.15 (0.09) kN and 0.08 (0.04) [%] for the reference parameters as the initial guess to 3.67 (2.46) kN and 1.25 (0.76) [%] for the initial guess that was the farthest away from the reference values (Table 3). Figure 3 summarizes the estimations of the ligament properties for the different initial guesses.

Small kinematic RMSEs were observed for all initial guesses. The translation error was smaller than 1 mm for all knee flexion angles, while both internal/external and varus/valgus errors were smaller than 1.2° for all knee flexion angles (Table 4). Furthermore, smaller kinematic RMSEs were found in this computational study for all initial guesses compared to previous experimental results [1,2,9]. Table 5 presents the kinematic RMSEs of previous cadaver studies and initial guess 4 from this study, which demonstrated the worst estimation for the ligament parameters.

**Figure 4:**
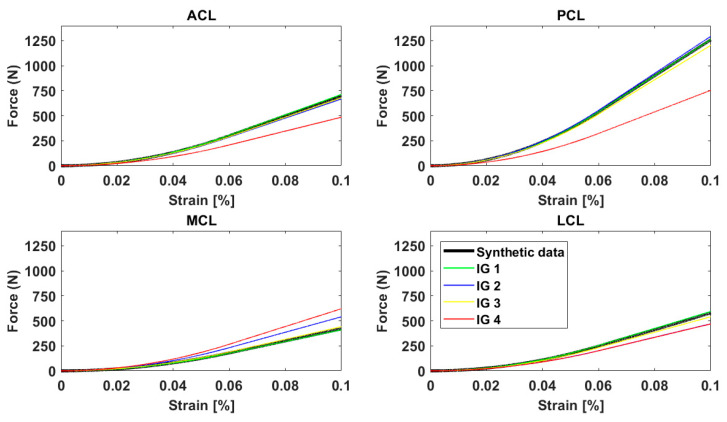
Ligament force–strain curves for the anterior cruciate (ACL), posterior cruciate (PCL), medial collateral (MCL) and lateral collateral (LCL) ligaments. The curves using the reference ligament parameters (synthetic data) and the optimized values for the different initial guesses (IG) are presented.

Similar loading patterns were observed for the optimized values obtained from initial guesses 1 and 3 and the synthetic data (Figure 4). The MCL force–strain curve for the optimized parameters using initial guess 2 was different compared to that using the reference parameters. Moreover, all force–strain curves were different for the optimized values with initial guess 4 and the synthetic data (Figure 4). These differences are highlighted by the ligament forces computed with models using the optimized parameters obtained with initial guesses 1 and 4 for the laxity tests included in the protocol (Table 6 and Table 7).

## 4. Discussion

The presented study investigated the influence of the initial guess on the estimation of ligament parameters via optimization procedures which aim to match simulations to kinematic data from laxity tests. We generated a synthetic data set using a computational model and literature values for the ligament parameters. Our synthetic data set was free of measurement errors and inaccuracies, and thus, we were able to investigate how the initial guess affects the computation of ligament properties unaffected by experimental errors. As expected, it was demonstrated that the initial guess influences the computation of ligament properties.

The laxity behavior of our model was within the physiological ranges reported in the literature (Table 2). The kinematic response of our model to simulated anterior and posterior drawer tests matched the respective experimental measurements [22,24]. Our simulations showed good agreement with measurements provided by Liu et al. [21] for internal and varus moments, but not for external moments. The loading scenarios that included external moments underestimated Liu et al.’s [21] measurements, but were in agreement with the kinematic measurements presented by Shultz and al. [20]. The valgus simulations showed a similar trend compared to the measurements reported by Wierer et al. [23]. Overall, these comparisons demonstrate that our model simulates the laxity profile of a random healthy individual.

The computations of the ligament parameters showed that the accurate prediction of ligament parameters is possible when an initial guess close to the true ligament parameters is provided. This was shown for initial guesses 1, 2 and 3, which had up to 1.1 kN and a 1.3% mean distance from the reference stiffness and reference strain, respectively. It should be noted, though, that only three initial guesses are not enough to define the problem’s basin of attraction. A study with many more variations in the initial guess is needed to generalize this observation and identify how close an initial guess should be to the true ligament parameters to ensure their accurate estimation. However, the reported accurate estimation of the parameters remains an encouraging finding as it supports the use of the presented procedures for the estimation of ligament parameters. This has great clinical significance as, when combined with experimental procedures, it enables the estimation of ligament parameters in an in vivo and noninvasive manner.

Our hypothesis for worse estimations of the ligament parameters when the distance of the initial guess from the reference values increased was supported. This most probably occurred due to the nature of this optimization problem that has many local minima. It was shown that the optimization process, at least when using the Complex optimization method, might provide a suboptimal solution with a set of ligament parameters that produces biomechanical behavior close to the target behavior. The Complex optimization was selected, as the preliminary results [14] showed better performance compared to the simulated annealing, which is commonly adopted [1,2,9] for such procedures. Future studies should investigate whether other optimizers, such as the genetic algorithm or particle swarm, could overcome this challenge.

Simulations performed with the optimized parameters obtained with initial guess 4 demonstrated that a set of ligament parameters different from the true ones can provide a similar laxity profile with translation differences of less than 1 mm and rotational differences of less than 1° compared to the reference kinematics. However, the optimized ligament parameters using initial guess 4 were, on average, 3.67 (2.46) kN for the stiffnesses and 1.25 (0.76) [%] for the reference strains, different compared to the respective true values. These differences in the parameters led to different loading patterns for the ligaments (Table 6 and Table 7). The observed differences in the ligament loading patterns are supported by previous studies, demonstrating that computational models are sensitive to changes in ligament parameters [33,34]. Future studies should investigate whether such differences in ligament loading could lead to altered conclusions about the design and/or the performance of clinical interventions such as braces, implants and surgical planning.

The present computational study demonstrated how challenging it is to compute ligament parameters accurately, even with no experimental uncertainties. In experimental set ups, such uncertainties might be introduced during the identification of application points, directions and magnitudes of forces, or application axes of moments and during the kinematic measurements. Moreover, inaccuracies might occur in the digitalization of the bones and the identification of the locations of the ligament insertions sites. Such uncertainties and inaccuracies can affect the predictions of the ligament parameters [13]. Even though we observed smaller kinematic errors in our study compared to studies where optimization procedures were employed combined with experimental measurements (Table 4), we obtained a set of ligament parameters that resulted in similar laxity behavior to the true behavior. This indicates that the previously predicted ligament values [1,2] could be different from the true values. Researchers have raised concerns that different combinations of ligament properties could provide similar target biomechanical behavior [1,2]. This was recently demonstrated in an experimental case study [12]. The findings of this computational study free of experimental inaccuracies confirms this concern, which should be further investigated.

This study has some limitations that should be mentioned. Firstly, this study was conducted on only one specimen using the four main knee ligaments, neglecting other structures such as capsular tissues and menisci. Furthermore, the implemented ligaments were simulated as single line elements without wrapping around bones, as it is physiologically. The single-element representation for each ligament neglects the different behavior the bundles of the same ligamentous structures have under knee joint motions [35]. Therefore, ligaments are often simulated as multiple linear elements or even as 3D continuum representations [10]. Furthermore, as the aim of our study was to compute joint laxity, the contact of the articular surfaces was modeled with a so-called rigid-to-rigid contact model, with the cartilage being modeled as a rather stiff material. To simulate accurate cartilage deformations, different approaches are needed, such as finite element analysis. Our computationally inexpensive choices kept the number of design variables to a minimum. This allowed us to explore different scenarios, which were sufficient to demonstrate the challenges of estimating ligament properties via optimization procedures.

## 5. Conclusions

The presented computational study showed that accurate ligament properties can be obtained when accurate laxity measurements and initial guesses close to the true values are provided. However, simulations with many more initial guesses are needed to define the problem’s basin of attraction. Additionally, the optimization procedure might result in suboptimal solutions with combinations of ligament parameters that provide behavior similar to the target biomechanical behavior. This is an alarming finding, and future research should aim to provide methodological procedures to overcome this challenge by exploring different optimization procedures.

## Figures and Tables

**Table 1:** The initial guess (IG) of the stiffness (k) in kN and reference strain (ε_r_) [%] of the ligaments used for each set and their mean distance from the reference values.

Sets	ACL		PCL		LCL		MCL		Mean Distance from Reference Values	
	k	ε_r_	k	ε_r_	k	ε_r_	k	ε_r_	k	ε_r_
Reference values	10.0	8.0	18.0	−4.0	6.0	2.0	8.3	4.0		
IG 1	10.0	8.0	18.0	−4.0	6.0	2.0	8.3	4.0	0.0	0.0
IG 2	9.5	7.5	17.5	−3.5	6.5	2.5	8.0	4.5	0.5	0.5
IG 3	9.0	7.0	17.0	−3.0	7.0	4.0	7.0	5.0	1.1	1.3
IG 4	8.0	4.0	8.0	4.0	8.0	4.0	8.0	4.0	3.6	3.5

**Table 2:** Experimental laxity measurements from the literature and simulated laxity values with our model for different loading scenarios.

Load Scenario (Study)	Magnitude	Knee Flexion	Experimental		Model
			Mean	sd	
Anterior force (Senioris 2017 [24])	134 N	30	5.6	3.1	5.7
Posterior force (Nau 2005 [22])	100 N	30	6.1	2.5	9.4
Internal moment (Liu 2014 [21])	5 Nm	30	21.0	2.8	23.0
Internal moment (Liu 2014 [21])	5 Nm	60	20.8	4.6	25.0
External moment (Shultz 2010 [20])	5 Nm	20	12.4	3.6	10.4
External moment (Liu (2014 [21])	5 Nm	30	17.7	2.7	7.0
External moment (Liu 2014 [21])	5 Nm	60	18.7	4.2	10.0
Varus moment (Liu 2014 [21])	10 Nm	0	2.5	1.1	1.9
Varus moment (Liu 2014 [21])	10 Nm	30	5.4	1.7	5.4
Varus moment (Liu 2014 [21])	10 Nm	60	6.3	1.6	7.0
Valgus moment (Wierer 2021 [23])	8 Nm	0	2.1	0.4	−0.2
Valgus moment (Wierer 2021 [23])	8 Nm	30	3.7	0.9	1.3
Valgus moment (Wierer 2021 [23])	8 Nm	60	4.4	1.2	2.4

**Table 3:** The mean error and standard deviation (sd) for the estimation of the stiffness (k) and reference strain (εr) parameters of the ligaments for each initial guess (IG).

Initial Guess	k (kN)		ε_r_ [%]	
	Mean	sd	Mean	sd
IG 1	0.15	0.09	0.08	0.04
IG 2	0.48	0.23	0.35	0.19
IG 3	1.05	0.67	1.16	0.90
IG 4	3.67	2.46	1.25	0.76

**Table 4:** The absolute kinematic error for the different sets of IGs and knee flexion angles.

Measurement	Anterior Translation (mm)			Internal Rotation (°)			Varus Rotation (°)		
Knee Flexion	0	30	60	0	30	60	0	30	90
Set 1	0.0	0.2	0.0	0.1	0.1	0.1	0.0	0.0	0.0
Set 2	0.2	0.4	0.3	0.2	0.1	0.1	0.0	0.0	0.1
Set 3	1.0	0.8	0.7	0.6	1.0	1.1	0.5	0.4	0.2
Set 4	1.0	0.7	0.5	0.9	1.0	0.4	0.6	0.5	0.5

**Table 5:** Kinematic differences between the simulated and experimental results for initial guess 4 and previously presented studies. Baldwin ^a^ refers to internal rotation and varus, while Baldwin ^b^ refers to external rotation and valgus.

Study (Knee Flexion)	Internal Rotation (°)		Varus Rotation (°)		Anterior Translation (mm)	
	Mean	sd	Mean	sd	mean	sd
Baldwin et al. [1] ^a^	2.6	0.3	0.6	0.4		
Baldwin et al. [1] ^b^	2.2	0.9	1.4	1.0		
Ewing et al. [2] 0°	3.2	2.2	1.7	1.3		
Ewing et al. [2] 20°			4.0	2.7		
Ewing et al. [2] 90°			4.3	2.9		
Harris et al. [8] 0°	4.9	0.7	1.2	0.4	2.0	1.2
Harris et al. [8] 15°	5.8	1.9	1.8	0.5	2.2	1.0
Harris et al. [8] 30°	3.5	1.1	1.5	0.5	1.5	0.7
Harris et al. [8] 45°	2.1	0.7	1.2	0.6	1.4	0.6
Harris et al. [8] 60°	4.1	1.4	1.7	1.2	1.8	0.8
IG 4. 0°	0.9		0.6		1.0	
IG 4. 30°	0.7		0.5		0.7	
IG 4. 60°	0.4		0.5		0.5	

**Table 6:** The maximum differences between the ligament forces computed with models using the synthetic data and the optimized parameters obtained with initial guess 4. The ligament forces for the anterior (ACL), posterior cruciate (PCL), medial (MCL) and lateral collateral (LCL) ligaments are presented per knee flexion angle.

Knee Flexion (°)	ACL Force (N)		PCL Force (N)		MCL Force (N)		LCL Force (N)	
	IG 1	IG 4	IG 1	IG 4	IG 1	IG 4	IG 1	IG 4
0	278	238	32	20	155	242	384	400
30	237	183	63	40	160	244	293	262
60	409	350	263	201	141	228	191	166

**Table 7:** The maximum differences between the ligament forces computed with models using the synthetic data and the optimized parameters obtained with initial guess 4. The ligament forces for the anterior (ACL), posterior cruciate (PCL), medial (MCL) and lateral collateral (LCL) ligaments are presented per loading scenario.

Load Scenario	ACL Force (N)		PCL Force (N)		MCL Force (N)		LCL Force (N)	
	IG 1	IG 4	IG 1	IG 4	IG 1	IG 4	IG 1	IG 4
Anterior force	373	322	65	40	95	176	63	55
Posterior force	153	142	212	241	99	182	42	29
Varus moment	271	247	124	165	88	161	85	93
Valgus moment	126	93	151	124	127	210	91	85
ER moment	237	183	263	201	114	193	67	70
IR moment	409	350	53	88	141	228	293	262

## Data Availability

The data presented in this study are available on request from the corresponding author.
